# Advancing food safety with biogenic silver nanoparticles: Addressing antimicrobial resistance, sustainability, and commercial viability

**DOI:** 10.1016/j.fochx.2025.102298

**Published:** 2025-02-20

**Authors:** Himanshu Jangid, Harish Chandra Joshi, Joydeep Dutta, Akil Ahmad, Mohammed B. Alshammari, Kaizar Hossain, Gaurav Pant, Gaurav Kumar

**Affiliations:** a School of Bioengineering and Biosciences, Lovely Professional University, Jalandhar, Punjab, India; b Department of Chemistry, Graphic Era (Deemed to be University), Dehradun 248002, Uttarakhand, India; c Department of Chemistry, College of Science and Humanities in Al-Kharj, Prince Sattam bin Abdulaziz University, Al-Kharj 11942, Saudi Arabia; d Department of Environmental Science, Asutosh College, University of Calcutta, 92, Shyama Prasad Mukherjee Rd, Bhowanipore, Kolkata 700026, West Bengal, India; e Department of Microbiology, Graphic Era (Deemed to be University), Dehradun, Uttarakhand, India; f Amity Institute of Microbial Technology (AIMT), Jaipur, Rajasthan, India

**Keywords:** Silver nanoparticles, Foodborne pathogens, Antimicrobial resistance, Green synthesis, Food safety

## Abstract

The escalating threat of antimicrobial resistance (AMR), particularly among foodborne pathogens such as *Escherichia coli*, *Salmonella enterica*, and *Listeria monocytogenes*, necessitates innovative solutions beyond conventional antimicrobials. Silver nanoparticles (AgNPs) have garnered significant attention for their broad-spectrum antimicrobial efficacy, ability to target multidrug-resistant strains, and versatile applications across the food sector. This review critically examines AgNPs' integration into food safety strategies, including their roles in antimicrobial food packaging, agricultural productivity enhancement, and livestock disease mitigation. Key advancements in eco-friendly synthesis methods, leveraging algae, agricultural byproducts, and microbial systems, are highlighted as pathways to address scalability, sustainability, and cost constraints. However, the potential risks of silver bioaccumulation, environmental toxicity, and regulatory challenges present significant barriers to their widespread implementation. By reviewing cutting-edge research, this review provides a comprehensive analysis of AgNP efficacy, safety, and commercial viability, proposing a roadmap for overcoming current limitations. It calls for collaborative, interdisciplinary efforts to bridge technological, ecological, and regulatory gaps, positioning AgNPs as a transformative solution for combating AMR and ensuring global food security.

## Highlights

• Biogenic AgNPs revolutionize control of foodborne pathogens and antimicrobial resistance.
• Advanced Green synthesis ensures sustainable and scalable AgNP applications in food safety.
• AgNPs enhance food packaging, processing, and agriculture by reducing contamination.
• Regulatory frameworks and eco-safeguards are key to AgNP adoption globally.

## 1 Introduction

Antimicrobial resistance (AMR) poses a grave threat to public health, food safety, and global economic stability. The World Health Organization (WHO) projects that by 2050, AMR could cause up to 10 million deaths annually, surpassing cancer as a leading cause of mortality (Salam et al., 2023). Foodborne pathogens such as *Salmonella enterica*, *Escherichia coli*, *Listeria monocytogenes*, and *Campylobacter jejuni* are particularly concerning, as they are responsible for significant morbidity and mortality while exhibiting increasing resistance to antibiotics (de Oliveira et al., 2024). Globally, the misuse of antimicrobials in agriculture is a primary driver of AMR in foodborne pathogens. An estimated 73 % of antimicrobials are used in livestock, often at subtherapeutic levels to promote growth and prevent disease, fostering the selection of resistant strains (The FAO Action Plan on Antimicrobial Resistance, 2021–2025, 2021). Studies have shown that *Campylobacter jejuni* exhibits resistance to fluoroquinolones in over 40 % of isolates worldwide, while *E. coli* has developed resistance to multiple antibiotics, including ciprofloxacin and ceftriaxone, threatening the efficacy of critical treatments (Mencía-Gutiérrez et al., 2024). Foodborne AMR infections impose a substantial health and economic burden. In 2019, Europe alone reported over 30,000 deaths linked to foodborne AMR infections, with total healthcare and productivity losses estimated at over €1.1 billion annually (“EU Action on Antimicrobial Resistance - European Commission,”, 2024). In the U.S., foodborne AMR infections are estimated to account for 400,000 cases annually, primarily attributed to *Salmonella* and *Campylobacter* (Samtiya et al., 2022). With traditional antibiotics losing their efficacy, silver nanoparticles (AgNPs) have emerged as a promising alternative. AgNPs exhibit broad-spectrum antimicrobial activity and are effective against multidrug-resistant (MDR) strains of *Listeria monocytogenes* and *E. coli* by disrupting cell membranes and generating reactive oxygen species (Chandraker & Kumar, 2024; Kah et al., 2023; More et al., 2023).

Despite their promise, the application of AgNPs in food systems faces challenges related to toxicity and environmental accumulation. Regulatory agencies, including the European Food Safety Authority (EFSA) and the United States Environmental Protection Agency (EPA), have developed strict guidelines for the safe use of nanomaterials in food applications (EFSA Scientific Committee et al. Markowska et al., 2018). The FAO has also emphasized the need for risk assessment frameworks to ensure the safe integration of AgNPs into food systems (FAO, 2014). This review critically evaluates the potential of AgNPs as antimicrobial agents against MDR foodborne pathogens. It examines their mechanisms of action, synergistic effects with antibiotics, and applications in food systems while addressing the regulatory, environmental, and safety challenges associated with their use. By reviewing the latest research and identifying gaps in knowledge, this paper aims to provide a comprehensive perspective on leveraging AgNPs to mitigate AMR in foodborne pathogens.

## 2 Mechanisms of action and efficacy of silver nanoparticles

**Fig. 1:**
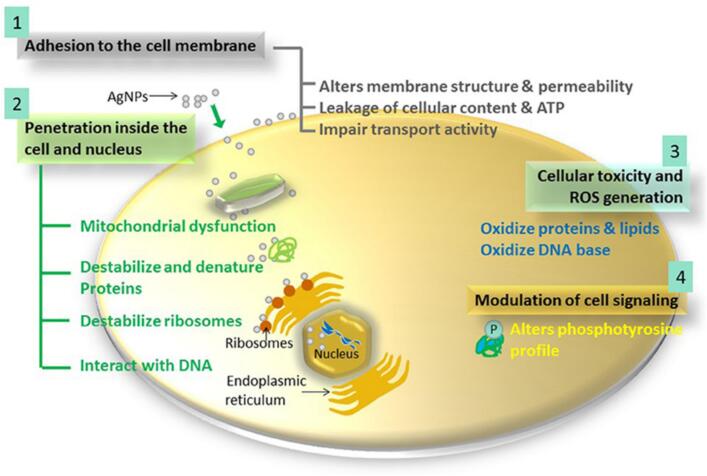
Mechanism of antimicrobial action of silver nanoparticle involving four key prominent routes taken from (Dakal et al., 2016).

Silver nanoparticles (AgNPs) constitute a significant player in overcoming multidrug-resistant pathogens. Their apparent antibacterial effectiveness stems from unique physicochemical properties that support multiplexed mechanisms of action, thus rendering them distinctly different from conventional antibiotics. Unlike antibiotics acting on a single pathway, AgNPs exploit a multitargeted approach, reducing the possibility of resistance development. The molecular mechanisms underpinning their efficacy and evaluate their relative effectiveness in comparison with conventional antimicrobials. The core action of AgNPs against bacteria is related to their membrane disruption activity. Due to their large surface-area-to-volume ratio and high surface reactivity, electrostatic interactions between AgNPs with the negatively charged bacterial membrane serve to structurally disrupt it. The increased membrane permeability and leakage from bacterial cytoplasmic contents are effective means for compromising bacterial viability. Recent research has demonstrated changes in morphologies due to silver nanoparticles in *Escherichia coli and Staphylococcus aureus.* This includes ripping off the membrane and cell shrinking as observed using electron microscopy (Summer et al., 2024). Besides these physical damages, reactive oxygen species like hydroxyl radicals and superoxide ions are formed. ROS leads to oxidative stress that disrupts the lipids, proteins, and nucleic acids; therefore, injuring the cells and leading to cell death. The ROS-mediated pathway is the most effective in disrupting biofilms, which are recognized for their high resistance to antibiotics (Juan et al., 2021). The next important mechanism of action is in the interaction of AgNPs with bacterial proteins and enzymes. By binding with the sulfur and phosphorus-containing functional groups, AgNPs inactivate important pathways of enzymatic functions that disrupt cellular respiration and energy production. This also causes a disturbance in protein folding, thus further limiting bacterial metabolic functions. Another important mechanism is DNA interaction. AgNPs enter the bacterial cell and interact with genetic material, inhibiting its replication and transcription. Such a mode of multidimensional disruption in the cell machinery explains the broad-spectrum activity of AgNPs against both Gram-negative and Gram-positive bacteria as depicted in Fig. 1 (Leão et al., 2024).

AgNPs have a few comparative advantages over common antibiotics. Unlike the common antibiotics mainly work through specific bacterial processes, AgNPs work in disturbing physical, oxidative, and biochemical processes. For example, according to the latest experimental studies, AgNPs showed an 85 % reduction of *Salmonella enterica* growth in contaminated poultry samples than the common antibiotics, ciprofloxacin (Dias de Emery et al., 2022). Most importantly, AgNPs demonstrate synergistic effects, making their efficacy increase up to 70 % with antibiotics. This synergistic effect promotes a reduction in dosages of required antibiotics that could reduce side effects and retard the growth of resistance (Nqakala et al., 2021). Apart from the inherent antimicrobial activity, the multi-functionality of AgNPs disrupts biofilms as well as interferes with quorum sensing - one of the most important ways by which bacteria coordinate their virulence and resistance. Biofilm inhibition is highly effective against pathogens such as *Pseudomonas aeruginosa* and *Listeria monocytogenes* for which the effectiveness of antibiotics is limited (de Lacerda Coriolano et al., 2020). In addition, their application in food safety has been promising, where silver nanoparticle-coated packaging materials extend the shelf life of perishable commodities by preventing microbial contamination (Mathew & Radhakrishnan, 2021; Rao et al., 2024). However, despite these positive features, the use of silver nanoparticles in applications poses certain challenges as well. Given the cytotoxic activity and persistence of AgNPs in the environment, safe synthesis and controlled application methods have been an interesting research area. The European Food Safety Authority and the United States Environmental Protection Agency have also outlined guidelines to reduce these risks, thereby allowing the safe harnessing of the benefits offered by AgNPs without compromising safety (Ahmad et al., 2024; EFSA Scientific Committee et al., 2018). In summary, the varied mechanisms through which AgNPs act and the high efficacy combined with probable synergism place them among those forces that are winning the battle against MDR pathogens. Potential concerns related to toxicity and environmental impact would be fully addressed before such capabilities find their way into clinical and food safety applications.

## 3 Advanced green synthesis techniques for silver nanoparticles: Sustainable innovations

Green synthesis techniques for silver nanoparticles (AgNPs) are gaining prominence due to their eco-friendly approach, reliance on renewable resources, and potential for large-scale production. Thus, silver nanoparticles (AgNPs) are currently being synthesized through green synthesis techniques, which are well accepted today as more environmentally friendly, renewable material-based, and feasible for mass-scale production (Fahim et al., 2024; Kiani et al., 2023; Nqakala et al., 2021). Approaches in the biological resources include marine algae, agricultural byproducts, and microbial systems to facilitate the reduction of silver ions into nanoparticles with minimal involvement of hazardous chemicals conventionally used in the fabrication of nanoparticles. Developments in the synthesis of plant-based and enzymatic methods offer new avenues for increasing efficiency, scalability, and environmental sustainability (Akhter et al., 2024; Abasi et al., 2022). Marine algae, both microalgae, and macroalgae, are emerging as potent bioresources for AgNP synthesis due to the abundance of bioactive compounds such as polysaccharides, phenols, and proteins that can act as natural reducing and stabilizing agents (Palanisamy et al., 2024). The synthesized AgNPs from various algae sources, including the *Laurencia papillosa* and *Sargassum myriocystum,* possess exceptional antimicrobial activities against human pathogens like foodborne pathogens, *Escherichia coli,* and *Staphylococcus aureus.* Such nanoscale metal particles are synthesized with low environmental hazards since they utilize the inherent reducing properties of algae while minimizing the toxic byproducts (Shanmugam et al., 2014). Marine algae also contribute to the economic feasibility, as these are ubiquitous, thus reducing the cost of feedstocks and less processing involved (Mukherjee et al., 2021). Agricultural waste that consists of fruit peels, husks, and crop residues, is an even greener alternative for nanoparticle preparation. These materials contain phytochemicals such as flavonoids and terpenoids that contribute to the bio-reduction of the silver ions. For instance, banana peels and sugarcane bagasse-based AgNPs possess good stability and efficient antimicrobial action and are appropriate for food packaging and agriculture (Ghosh et al., 2017). Considering the principles of circular economy, the strategy of conversion of agricultural crop waste into useful nanomaterials reduces the pollution that would have otherwise been released from the environment and also decreases the carbon footprint associated with nanoparticle fabrication (Priyadarshini et al., 2023). Microbial synthesis has developed into a novel method wherein microbes associated with bacteria, fungi, and algae function as natural bioreactors for the production of silver nanoparticles (AgNPs). These microorganisms secrete enzymes and metabolites that facilitate the reduction of silver ions, thereby allowing for precise control during the synthesis process. For example, *Bacillus subtilis* and *Aspergillus niger* have been genetically modified to produce uniform and remarkably stable nanoparticles, while microbial fermentation processes promote scalability (Bahrulolum et al., 2021). Algal-associated microbes have also been targeted for their unique metabolic pathway, enhancing the stability of nanoparticles and reducing the synthesis time (Hamida et al., 2022).

**Fig. 2:**
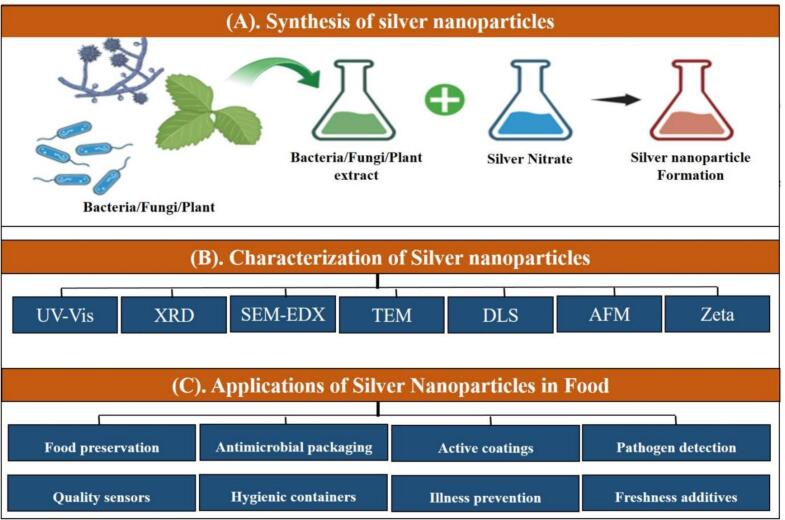
(A) Schematic representation of the mechanism biogenic synthesis of silver nanoparticles (B) Characterization and (C) Applications.

Enzymatic as well as plant-based synthesis have immensely enriched the domain. Biological system-derived enzymes provide very effective control over the sizes and shapes of nanoparticles, a very crucial aspect while trying to achieve maximum antimicrobial efficacy. The plant extracts neem, tea leaves, and moringa have been well established to be promising in producing AgNPs showing maximum biocompatibility together with an eco-friendlier nature (Vanlalveni et al., 2021). The methodologies presented are unnecessary for external stabilizing agents and follow the principles of green chemistry by using renewable resources (as shown in Fig. 2).

Recent advancements in biosynthesis have focused on genetically modified algae and microbial systems to enhance the scalability and efficiency of silver nanoparticle (AgNP) production. For instance, genetically engineered *Desmochloris edaphica* has been used to synthesize stable AgNPs with significant antimicrobial activity against pathogens like *Staphylococcus aureus* and *Shigella flexneri* (Hamida et al., 2024). Recent advancements in artificial intelligence (AI) have significantly enhanced the biosynthesis of silver nanoparticles (AgNPs) by optimizing reaction parameters and improving scalability. For example, Bayesian optimization has been successfully applied to fine-tune synthesis variables, such as temperature, reagent concentrations, and reaction time, achieving highly efficient nanoparticle production with precise optical properties (Yoo et al., 2023). Additionally, AI-based interaction modeling has enabled the identification of phytoconstituents crucial for AgNP formation, enhancing the reproducibility and stability of the biosynthesis process (Naghizadeh et al., 2021). These advancements not only streamline the production process but also facilitate large-scale applications, making AgNP synthesis more cost-effective and sustainable. Additional advancements include hybrid approaches that bio-chemical routes combine to achieve the greatest efficiency and functionality. For instance, the merger synthesis using algae and microwave-assisted methods accelerates production without compromising environmental sustainability (Khan et al., 2022; Tarannum et al., 2019; Torabfam & Yüce, 2020). Microbe/plant-based bioengineering systems with genetically engineered modification may also enhance the yield and reproducibility of the nanoparticles. Advanced green synthesis techniques are applied toward overcoming critical challenges relating to scalability, environmental impact, and cost-effectiveness. Marine algae and agricultural wastes are readily available and relatively inexpensive resources. On the other hand, accuracy and reproducibility are achieved through microbial and enzymatic methods. Such technologies can be added with nanofabrication and real-time monitoring systems that will make AgNPs more relevant for controlling foodborne pathogens as well as other crucial fields. This type of sustainable innovations taken by the industries can connect with the global sustainability goals by exploiting their unique characteristics in AgNPs for various purposes in food safety, agriculture, and biomedicine. The significant attention given to innovative methods of green synthesis places AgNPs at the very center of sustainable nanotechnology, which could be applied to nearly any field and simultaneously mitigates critical environmental and economic problems. Further, Table 1 mentions the biosynthesis of silver nanoparticles using different microorganisms (Bruna et al., 2021).

**Table 1:** . Biosynthesis of silver nanoparticles using different microorganisms.

**Microorganism**	**Key Findings**	**Mechanism**	**Particle Size**	**Morphology**	**Reference**
*E. coli*	Produced AgNPs in periplasmic space under anaerobic conditions	Biological reduction of Ag + ions, role of c-type cytochromes	20.8 nm	The zero-valent metallic silver lattice	(Solís-Sandí et al., 2023)
*Rhodococcus* sp.	Synthesis of AgNPs by reducing silver nitrate	Cell enzyme systems may participate in the synthesis	20–50 nm	Spherical	(John et al., 2022)
*Aspergillus tubingensis*	Fungal production of stable silver hydrosol	Enzymatic reduction of metal ions	35 ± 10 nm	Stabilized by proteins secreted by the fungus	(Fonseca et al., 2022)
*Cinnamomum camphora leaf*	Fabrication of AgNPs at ambient temperature	Reduction of silver ions or chloroaurate ions, protective biomolecules	19.57 nm	Triangular or spherical	(Li et al., 2021)
Mango peel extract	Synthesis from aqueous silver nitrate using Mango peel extract	Biochemical mechanism involving FTIR	2.5–6.5 nm	Crystalline, face-centered cubic symmetry	(Cheng et al., 2021)
*Bjerkandera* sp. *R1*	Intracellular reduction of Ag + ions by fungus biomass	Reduction by enzymes in the cell wall membrane	10–100 nm	Formed below the cell wall surface	(Osorio-Echavarría et al., 2021)
*Plectonema boryanum UTEX 485*	Cyanobacterium reacted with AgNO3	Metabolic processes and organics from dead cyanobacteria	Up to 200 nm	Spherical and octahedral	(Behera et al., 2024)
*Pelargonium x hortorum*	Rapid reduction of silver ions leading to AgNPs	Faster than fungal methods, possibly enzyme-mediated	16 to 40 nm	Assembled into quasilinear superstructures	(Behera et al., 2024)
*Syzygium cumini fruit extract*	Synthesis and anti-cancer activities of AgNPs	Flavonoids are responsible for the reduction and stabilization	10–15 nm	Not specified	(Mittal et al., 2014)
*Chlamydomonas reinhardtii*	Biosynthesis of SNPs by unicellular algae	Cellular oxidoreductase proteins control	5 ± 1 to 35 ± 5 nm	Not specified	(Shahriari Ahmadi et al., 2016)
*Penicillium verrucosum*	Biosynthesis of AgNPs with antifungal activity	Reaction with AgNO3	10–12 nm	Polydisperse	(Yassin et al., 2021)
*Aspergillus sydowii*	Green synthesis of AgNPs	Reduction of Ag + ion, enzyme-mediated extracellular reaction	24 nm	Spherical, polydispersed	(Wang et al., 2021)
*Aspergillus flavus F5*	Synthesis of spherical metallic AgNPs	Extracellular synthesis process	12.5 nm	Spherical	(Fouda & Awad, 2022)
*Aspergillus terreus*	Extracellular synthesis of AgNPs	Probable extracellular synthesis mechanism	7–23 nm	Spherical	(Lotfy et al., 2021)
*Solanum tuberosum peel* aqueous extract.	The functional group capped AgNPs	Green biosynthetic method	3.91 to 27.07 nm	Crystalline silver, amorphous iron	(Xu et al., 2023)
*Garcinia mangostana leaf extract*	Eco-friendly synthesis of AgNPs	Reduction by leaf extract	35 nm	Not specified	(Karthiga, 2018)

## 4 Antagonistic activity of silver nanoparticles against foodborne pathogens

The rise of multidrug-resistant (MDR) foodborne pathogens, such as *Salmonella enterica*, *Escherichia coli*, and *Listeria monocytogenes*, has necessitated the exploration of alternative antimicrobial strategies. One such alternative that attracted much interest due to its aggressive antagonism toward these pathogens is silver nanoparticles (AgNPs). What is unique in the antimicrobial performance of AgNPs is empirical data, synergistic use together with antibiotics, and potential integration into food safety policies. Silver nanoparticles display high antimicrobial activity against a broad spectrum of both Gram-negative and Gram-positive microorganisms (Anees Ahmad et al., 2020; Caniça et al., 2019; Maniah et al., 2024). Gram-negative bacteria including *E. coli* and *Pseudomonas aeruginosa* show increased susceptibility to AgNPs due to their relatively thinner peptidoglycan layer and less strengthened cell wall structure. However, studies point out that AgNPs synthesized via advanced biogenic routes could effectively transcend the structural barriers of Gram-positive pathogens like *Staphylococcus aureus* (Radzig et al., 2013). AgNPs prepared from the use of *Sargassum vulgare* exhibited inhibition zones of 23 and 38 mm against *Pseudomonas aeruginosa, Klebsiella pneumoniae*, and *E. coli* at a 100 μL concentration that outmatches ordinary antibiotics in similar studies (Hamouda & Aljohani, 2024). AgNPs have recently been discovered to be mixed with conventional antibiotics that let them produce advanced therapy. Higher antibacterial efficiency has been demonstrated through the conjugation of these with ciprofloxacin or amoxicillin, significantly lowering the MIC levels to inhibit MDR pathogens (Muddassir et al., 2022). Recent advancements highlight the ability of AgNPs to disrupt biofilm-forming cells effectively, with minimal biofilm eradication concentrations (MBEC) reported between 0.125 and 0.25 mg/mL. When combined with antibiotics, AgNPs further enhance biofilm disruption, demonstrating significant potential to combat biofilm-associated infections caused by MDR pathogens (Masadeh et al., 2024). For instance, AgNPs derived from the bacterium *Pseudoduganella eburnea* had its lowest MIC at 6.25 μg/mL against *Pseudomonas aeruginosa* and 3.12 μg/mL against *Staphylococcus aureus* when conjugated with ciprofloxacin (Md. A. Huq, 2020). This synergistic approach increases bactericidal activity while offering the potential to reduce the doses to a minimum, thereby reducing side effects and slowing resistance development. The above technique of biosynthesis was discussed, but its relevance to performance in antimicrobial systems requires further elaboration. Hence, AgNPs synthesized using fungi exhibited high stability and homogeneity by which they have demonstrated increased antimicrobial activity. Reports state that *Fusarium oxysporum*-derived AgNPs were found to have strong inhibitory activity against *Listeria monocytogenes* and *Salmonella enterica* with inhibition zones over 20 mm at lower concentrations (Ilahi et al., 2022).

Methods of synthesis through algae are recently in vogue as these methods can prepare nanoparticles with controlled size and have high antibacterial potency. The studies reported 22 mm of antibacterial activities for *E. coli* and 20 mm of *Bacillus cereus* for AgNPs prepared from *Oscillatoria limnetica* cyanobacteria (Hamouda et al., 2019a). Interestingly, with AgNPs, along with antibiotics, the inhibition zone was enlarged to 26 mm and established the possibility of integrated approaches (Thomas & Nair, 2014). Besides promising in vitro efficacy, applications of AgNPs in food safety have indeed been very exciting. The coatings and packaging material embedded with AgNPs have successfully prevented microbial contamination in perishable foods whose shelf-life has been extended up to 50 %. Incorporation of AgNPs into sanitizing systems for food processing surfaces has been effective in reducing the burden of bacteria and biofilm formation in particular against species of *Salmonella* and *Campylobacter* (Istiqola & Syafiuddin, 2020; Sharma et al., 2023). Additionally, green synthesis techniques, such as lignin-capped AgNPs, provide an eco-friendly alternative for producing nanoparticles with high efficacy against MDR bacteria while maintaining low cytotoxicity. These nanoparticles have shown success in in vivo infection models, suggesting their viability for integration into food safety and healthcare applications (Pletzer et al., 2021). However, there are several challenges that the AgNPs pose in terms of real-world application. For example, the toxicity of these nanoparticles, persistence in the environment, and regulatory compliance need to be addressed immediately. Advances in encapsulation technologies and biopolymer coatings are explored as part of the way forward in addressing these challenges. For example, reduced cytotoxicity combined with retained antimicrobial activity has been documented in biodegradable composites of AgNPs, opening wide a route toward sustainable usage in food systems (Pandey et al., 2020). Silver nanoparticles pose a plausible solution to the ever-increasing MDR threat posed by the growing foodborne pathogens, most particularly those synthesized via biogenic pathways (as shown in Table 2). AgNPs provide wide protection against antibiotic resistance due to their strong antimicrobial activity and synergy with antibiotics but will require additional studies to determine their long-term safety and environmental impact before applying them on a large scale. Advances in encapsulation technologies using biodegradable materials have further reduced the cytotoxicity of AgNPs while preserving their antimicrobial effectiveness, paving the way for safer and more sustainable large-scale applications (Yadav & Tare, 2024).

**Table 2:** . Antagonistic activity of biogenic silver nanoparticles (AgNPs) against foodborne pathogens.

Species Name	Types	Antagonistic Activity	Observation	Reference
*E. coli*	Bacteria	*Corynebacterium diphtheriae*	Silver nanoparticles coated with antibiotics show improved inhibition zones.	(Lee et al., 2019)
*Thermophilic Bacillus species*	Bacteria	*Salmonella typhi E. coli*	Zone of inhibition: 22 mm.	(Deljou & Goudarzi, 2016)
*Pseudoduganella eburnea MAHUQ-39*	Bacteria	*S. aureus* and *Pseudomonas aeruginosa*	MICs: 3.12 μg/mL (*S. aureus*) and 12.5 μg/mL (*P. aeruginosa*); MBCs: 6.25 μg/mL and 25 μg/mL.	(M.A.Huq, 2020)
*Chlorella vulgaris*	Algae	*S. aureus*	Inhibition was observed at 50 μg/mL.	(Soleimani & Habibi-Pirkoohi, 2017)
*Sargassum wightii*	Algae	*S. aureus, Bacillus rhizoids, E. coli, P.aeruginosa*	Inhibition zone: 8–15 mm, varying with concentration (20–50 μL).	(Rajivgandhi et al., 2021)
*Sargassum polycystum*	Algae	*P.aeruginosa, E.coli, S.aureus*	Zone of inhibition: 23 mm.	(Mandal et al., 2023)
*Turbinaria conoides*	Algae	*S. liquefaciens, Aeromonas hydrophila*	Inhibition zone: 32 mm at 100 μL.	(Oktaviani et al., 2019)
*Oscillatoria limnetica*	Algae	*E. coli B.cereus*	Inhibition zone: 38 mm at 100 μL.	(Hamouda et al., 2019b)
*Rice Starch*	Biomolecule	*S. aureus Streptococcus mutans*	MIC: 5.7 × 10^−12^ mol/L.	(Abbaszadegan et al., 2015)
*Ficus benghalensis*	Plant	Dental pathogens	Inhibition zones: 15 mm (*S. mutans*) and 18 mm (*L. acidophilus*) at 250 μg.	(Manikandan et al., 2017)
*Lysiloma acapulcensis*	Plant	*S. aureus, E. coli* and *P. aeruginosa.*	Antibacterial activity observed.	(Garibo et al., 2020)
*Phyllanthus emblica*	Plant	*Acidovorax oryzae*	Inhibition zones: 18 mm (*E. coli*), 16 mm (*S. aureus*), and 15 mm (*P. aeruginosa*).	(Khan et al., 2013)
*Phyla dulcis*	Plant	*Salmonella typhimurium, E. coli, S. aureus, and Listeria monocytogenes*	Inhibition: 10–12 mm zone.	(McMurray et al., 2020)
*Cucumis prophetarum*	Plant	*S. aureus, S. typhi*	Inhibition observed against *S. typhi*.	(Hemlata, 2020)
*Glycyrrhiza Glabra Amphipterygium adstringens*	Plant	*Enterococcus faecalis*, *Candida albicans*	At 1 mM: 78 % fungal growth inhibition (AgNPs).	(Rodríguez-Luis et al., 2016)
*Pu-erh tea leaf extract*	Plant	*Salmonella typhimurium, Klebsiella pneumoniae*	MIC for AgNPs: 3.9–7.8 μg/mL.	(Loo et al., 2018a)
*Murraya koenigii leaves*	Plant	*S. aureus* *E. coli*	MIC: 32 μg/mL (*MRSA/MSSA*), 32–64 μg/mL (ESβL-*E. coli*).	(Qais et al., 2019)
*Caltropis procera*	Plant	*Vibrio cholerae* *E. coli*	Ag-NPs and ZnO-NPs show antibacterial activity.	(Salem et al., 2015)
*Eriobotrya japonica leaf extract*	Plant	*E. coli,* *S.aureus*	AgNPs show stronger antibacterial effects against *S. aureus*.	(Vanlalveni et al., 2021)
*Fusarium scirpi*	Fungi	*Escherichia coli*	MIC: 25 mg/mL against planktonic *UPEC* cells.	(Rodríguez-Serrano et al., 2020)
*Penicillium polonicum*	Fungi	*Acinetobacter baumanii*	Inhibition zone: 21.2 ± 0.4 mm.	(Neethu et al., 2018)
*Aspergillus terreus Paecilomyces lilacinus Fusarium* sp.	Fungi	*S. aureus,* *S. enterica* and *Streptococcus pyogenes*	Inhibition zones: 14–20 mm.	(Choi & Ahsan, 2022)
*Fusarium acuminatum*	Fungi	*S. aureus,* *S. typhi,* *S. epidermidis* and *E. coli*	Inhibition zones are higher than antibiotics, showing superior antimicrobial activity.	(Durán et al., 2016)
*Penicillium notatum*	Fungi	*E. coli,* *Salmonella typhimurium* and *Enterobacter aerogenes*	Clear distinction in antimicrobial effectiveness between AgNPs from *P. funiculosum* GS2 and *A. solani* GS1, based on inhibition zone.	(Singh et al., 2014)
*Aspergillus niger and aspergillus terrus*	Fungi	*S. aureus* and *E. coli*	MRSA showed a significant zone of inhibition (20 mm) with AgNPs, while MRSE showed a slightly smaller inhibition zone (19 mm).	(Barakat & Gohar, 2012; Nanda & Saravanan, 2009)

## 5 Comparative analysis of silver nanoparticles and traditional antimicrobials in food and agriculture

**Fig. 3:**
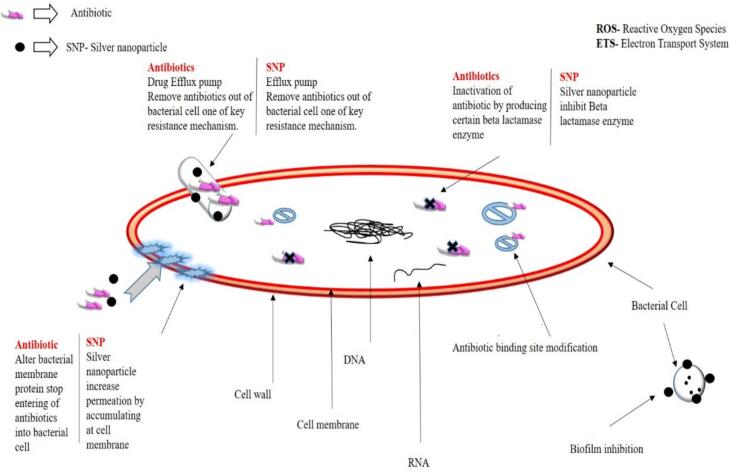
Comparative representation of antibiotic resistance mechanism and silver nanoparticle antimicrobial action mechanism.

Silver nanoparticles (AgNPs) are increasingly gaining recognition as a highly effective alternative to traditional antimicrobial agents, such as antibiotics and biopesticides, within the food and agricultural systems. They have broad-spectrum activities, multiple mechanisms of action, and a low propensity for resistance development. Mechanisms of action for AgNPs are different from traditional therapies since they do not, like antibiotics targeting particular biochemical pathways, exhibit several mechanisms of antimicrobial activity, including alterations in membrane integrity, ROS generation, and interference with DNA. This makes them especially effective against MDR pathogens. For instance, AgNPs have been shown to have even higher efficacy in the reduction of *Escherichia coli* and *Salmonella* on produce by up to 99.9 %, surpassing chlorine-based disinfectants used in conventional food processing (Loo et al., 2018b). Similarly, AgNP-based coatings in aquaculture demonstrated even greater efficacy than antibiotics in controlling biofilm-forming bacteria such as *Vibrio* spp., improving survival rates in shrimp farms (Elayaraja et al., 2021). AgNPs exhibit synergistic effects when combined with traditional antimicrobial agents. A recent study demonstrated that AgNPs reduced bacterial contamination in food products by 85 % while extending shelf life by up to 30 % (Zorraquín-Peña et al., 2020). Moreover, the combination of AgNPs with conventional antibiotics has shown synergistic effects, enhancing antimicrobial efficacy by up to 70 % in laboratory settings (Alotaibi et al., 2022). These findings underscore their potential as a tool for addressing AMR in foodborne pathogens. It was reported that the blend of AgNPs with antibiotics, including tetracycline and ciprofloxacin yielded a significant decrease in bacterial resistance along with increased overall effectiveness of antimicrobial activity (Ipe et al., 2020). Further, the combination of AgNPs with hydrogen peroxide has also been effective compared to the treatment obtained individually with MDR pathogens (Ipe et al., 2020). Although biopesticides and antibiotics are typically associated with problems about persistence in the environment, green synthesized AgNPs are more biodegradable and environmentally friendly. The agricultural applications of AgNPs, including pesticides made from agricultural biomass have been found to have lesser environmental toxicity than chemical pesticides (Wolny-Koładka et al., 2022). Further, Fig. 3 illustrates **a** comparative representation of the antibiotic resistance mechanism and silver nanoparticle antimicrobial action mechanism modified and adapted from (Bruna et al., 2021; Shaikh et al., 2019).

The cost-benefit analysis has shown that the greater initial production costs of AgNPs are more than the conventional antimicrobials, but long-term efficacy and reduced application frequencies make it economic in the long term. For example, AgNP-based coatings provide longer-lasting properties that give protection against antibiotics or pesticides, thus reducing the need for frequent reapplications (Zhou et al., 2017). Perhaps most importantly, the use of agricultural by-products in green synthesis methods has reduced the production cost by up to 30 % (Saratale et al., 2018). This enhances scalability and availability for various applications. There are still obstacles. Risks of toxicities of Ag ions to non-target species, coupled with the danger of adaptive microbial response to the abuse of AgNPs, thus demand careful attention. The regulatory framework assessing silver migration into food and environment needs further clarification for safety (Zhou et al., 2017). Despite these limitations, AgNPs are considered to be a sustainable alternative that holds vast potential for supplementing or replacing traditional antimicrobials in combating pathogens causing foodborne and agricultural diseases. Adoption of AgNPs would necessitate further research, especially into optimizing green synthesis while improving cost efficiency and developing sound regulation regimes. It would then be able to unlock the real potential of AgNPs as the foundation stone of antimicrobial strategies in food and agriculture.

## 6 Environmental and ecotoxicological impacts of silver nanoparticles in agriculture and ecosystems

With the rapid growth in the application of silver nanoparticles (AgNPs) in agricultural applications and food chains, the environmental concerns are immense mainly about the ecotoxicological impacts of these particles on soil, aquatic ecosystems, and microbial communities as mentioned in Table 3 (McGillicuddy et al., 2017). AgNPs have significant antimicrobial properties but unprecedented effects such as interference in nutrient cycling patterns and accumulation through food webs require extensive research studies. AgNPs, which are added to agricultural soils in the form of fertilizers, pesticides, or biosolids, can potentially disrupt critical microbial ecosystems involved in nutrient cycling. Exposure to AgNPs has been implicated in reducing both soil microbial diversity and enzymatic activity, with long-term implications for soil fertility and ecosystem imbalances; exposure to AgNPs has also been implicated in inhibiting the key soil bacteria *Nitrosomonas* and *Nitrobacter*, which play significant roles in nitrogen fixation and nitrification (McGee, 2020). Results in this study indicate that soil systems are susceptible to contamination by nanoparticles. Agricultural runoff disperses AgNPs into the water ecosystem, thus posing a significant risk to aquatic ecosystems. Once dispersed into the aquatic ecosystem, it can dissolve into ionic silver, Ag+, which has a highly toxic effect on aquatic life. It has been reported that the AgNPs interfere with the respiration and reproductive activities of *Daphnia magna* and zebrafish (McGee, 2020). Moreover, their propensity to circumvent filtration mechanisms at wastewater treatment plants increases contamination further down the pipeline, hence impacting larger ecological systems (Guo et al., 2019).

**Table 3:** . Environmental and ecotoxicological impacts of silver nanoparticles in agriculture and ecosystems.

**Category**	**Impact/Observation**	**Key Examples**	**Quantitative Data**	**Mitigation Strategies**
**Soil Ecosystems**	Reduction in microbial diversity and nutrient cycling disruption	AgNPs inhibited nitrifying bacteria (*Nitrosomonas* and *Nitrobacter*).	25 % reduction in soil nitrification rates after 30 days of exposure (Zhang et al., 2021)	Develop slow-release formulations and bio-inert coatings.
**Aquatic Systems**	Toxicity to aquatic organisms (*Daphnia magna*, zebrafish)	Exposure caused respiratory and reproductive impairments.	70 % reduction in reproductive success in *Daphnia* at 0.01 mg/L AgNP exposure (Park et al., 2021)	Use advanced nanoparticle filtration systems in wastewater treatment.
**Runoff and Sediment Contamination**	Increased silver concentrations in runoff and sediments	Paddy fields treated with AgNP fertilizers showed downstream contamination.	1.5 ppm silver was detected in runoff water (Park et al., 2021)	Establish buffer zones around treated areas to limit runoff.
**Bioaccumulation in Plants**	Accumulation of AgNPs in edible plant tissues	Silver residues are found in lettuce and wheat grown with AgNP fertilizers.	0.25 mg/kg silver in lettuce leaves (safe limit: 0.1 mg/kg) (Waalewijn-Kool et al., 2014)	Minimize AgNP use in direct fertilizers; implement foliar application.
**Food Chain Transfer**	Bioaccumulation in trophic levels	Detected in aquatic macrophytes, transferring to higher organisms.	Silver content increased 3-fold in fish exposed to contaminated macrophytes (Palácio et al., 2020)	Promote green synthesis methods to reduce ecotoxicity.
**Toxicity in Sediments**	Persistent toxicity affecting benthic organisms	Long-term sediment exposure led to mortality in soil arthropods (*Folsomia candida*).	30 % increase in mortality rates after 28 days (Waalewijn-Kool et al., 2014)	Deploy AgNP encapsulation technologies to minimize exposure.
**Wastewater Impact**	Ineffectiveness of conventional wastewater treatment methods	AgNPs bypassed filtration systems, contaminating downstream ecosystems.	35 % of AgNPs detected in downstream water (Waalewijn-Kool et al., 2014)	Develop AgNP-specific filtration and recovery systems.
**Algal Growth Suppression**	Reduced primary productivity in contaminated water bodies	Exposure affected algal photosynthesis and biomass accumulation.	40 % decrease in algal productivity at 0.05 mg/L AgNP exposure (Bakir et al., 2024)	Adopt eco-friendly production methods to reduce toxicity.

The other critical point is the bioaccumulation of AgNPs in plants and animals. AgNPs are expected to be absorbed into crop tissues via the application routes of fertilizers or foliar sprays and thereby ingested through edible parts, thus posing risks of human exposure through the food chain (Li et al., 2017). Studies on lettuce and wheat exposed to AgNP-enriched fertilizers revealed significant residues of silver in the crops, which consequently raised concern over the chronic exposure potential from these for human health (Wu et al., 2020). Case studies highlight the environmental persistence of AgNPs. For example, it has been reported that sewage sludge applied in agricultural settings can alter microbial communities and decrease enzymatic activity in soils. Furthermore, AgNP-enriched fertilizers used in the cultivation of rice paddies generated runoff, causing an increase in silver concentration in the targeted nearby water bodies with impacts on algal and invertebrate populations (Ottoni et al., 2020). Hence, sustainable strategies are inevitable in addressing these environmental risks. Green synthesis methods using either plants or microbes preclude the interference of reactive byproducts, minimizing inherent AgNP toxicity. Further restrictions on the bioavailability of AgNPs in ecosystems include controlled release systems such as biodegradable polymers encapsulating AgNPs. In addition, stronger regulations will be required to control responsible application and appropriate wastewater management that will minimize dispersal into the environment. Though the merits of AgNPs for agro-control of pathogens cannot be avoided, they require sustainability in their environment. The utility will have to be weighed with environmental protection through interdisciplinary research with strict oversight for sustainability without compromising their ecological footprint.

## 7 Emerging application of biogenic silver nanoparticles in food and agriculture

### 7.1 Emerging applications of silver nanoparticles in food packaging to control foodborne pathogens

Silver nanoparticles or AgNPs have emerged as the newest ingenuity in food packaging due to the unprecedented antimicrobial properties in the preservation of foods by the extension of perishable products. Nanoparticles work by making holes in the microbial cell membrane and allowing the release of silver ions, which interfere with the cell structure and cellular metabolism and lead to the killing of pathogens (Carbone et al., 2016; Kumar et al., 2021). It has been proven that PLA films doped with AgNPs can decrease the micro populations of *Salmonella enterica* and *Escherichia coli* by more than 99 % after a 48-h duration (Duda-Chodak et al., 2023). In such a regard, the incorporation of AgNPs into polyethylene films also neutralizes *Listeria monocytogenes* and is considered one of the most significant risks involved in ready-to-eat food products (Markowska et al., 2018). Some recent examples of innovations in AgNP-based food packaging include biodegradable films such as bacterial cellulose films combined with PVA films that can, together with extended antimicrobial activity, achieve environmental sustainability (Dairi et al., 2019). According to Begum et al. (2022), multilayered films with natural additives, such as essential oils or plant extracts, can hybridize silver nanoparticles (AgNPs) and achieve synergism in the fight against bacterial and fungal pathogens (Begum et al., 2022). Multi-layered films that embed AgNPs in their outer layers offer antimicrobial efficacy in targeted manners while inhibiting the migration of silver to food products; therefore, safe and effective (Kostic et al., 2019). Although AgNPs have been considered more potential to be applied in food packaging, their safety, regulatory approval, and environmental sustainability are still very crucial challenges. Studies on migration into foods show that the release of silver ions is material dependent on the package, characteristics of food, and storage conditions, which still raises the toxicity concern. According to Istiqola & Syafiuddin, 2020, regulatory bodies, including the FDA and EFSA, have provided regulations to standardize testing on safety. Moreover, trust from the public concerning the use of nanotechnology for food necessitates labeling and education of the consumers to change their perception (He et al., 2019; Ranjan et al., 2019).

However, environmental issues remain predominant to some extent because silver ions from spent packaging may survive in ecosystems and interfere with microbial diversity and nutrient cycling. On the contrary, there exists a current interest in AgNPs prepared through green synthesis techniques using plant-based precursors obtained from agricultural wastes; hence they are economically as well as environmentally friendly (Rodríguez-Félix et al., 2022). Future innovation breakthroughs will be achieved on the redetermination of strategies for food safety in terms of smart packaging with AgNPs and biosensors that can sense pathogens in real-time and hybrid nanocomposites with multifunctional properties. The best harvest of AgNPs to successfully control foodborne pathogen incidence necessitates greater-order interdisciplinarity collaboration between material scientists, microbiologists, and regulatory agencies. AgNP-based food packaging is one of the frontiers of technology for food safety, and it presents a sustainable solution for the effective mitigation of foodborne infections. The technology should, therefore advance toward wider applications by overcoming barriers associated with regulatory, safety, and environmental concerns through continued research and innovations.

### 7.2 Emerging applications of silver nanoparticles in food processing to control foodborne pathogens

Silver nanoparticles (AgNPs) have gained significant application in food processing because of their intrinsic antimicrobial properties, which may be required when handling food-borne pathogens and ensuring food safety. Their use in developing biosensors and antimicrobial coatings has transformed food processing because it reduces microbial loading on various surfaces and allows for real-time monitoring of pathogens. These developments are significant in controlling recurrent outbreaks by pathogens such as *Salmonella* spp.*, Escherichia coli,* and *Listeria monocytogenes* commonly contaminating foods at various points in processing and handling. AgNP-based antimicrobial coatings on food contact surfaces prevent the development of biofilms, which is the biggest challenge in the food-processing environment. The biofilms formed from the presence of bacteria such as *Listeria monocytogenes* have been proven to be resistant to conventional cleaning and result in pervasive contamination. It is known that the coating of AgNPs inhibits the development of biofilms by preventing adhesion and cellular structure formations. Rivera-Mendoza also found that incorporating AgNPs into the surfaces of processing equipment in poultry processing controlled *Campylobacter jejuni,* a common foodborne illness agent at greater than 90 % bacterial load reduction (Rivera-Mendoza et al., 2024a). Similarly, the long-term hygiene feature of coatings on equipment for the processing of meat and seafood stainless steel is provided with durable antimicrobial activity. Besides coating, AgNPs are an integral part of developing advanced biosensors for early detection of foodborne pathogens. The properties of optics and electronics of AgNPs enhance the sensitivity and specificity of these biosensors, which allows real-time analysis at a low concentration. For instance, AgNP-enhanced biosensors were designed to detect bacterial endotoxins as well as toxins from *E. coli* and *Staphylococcus aureus* in just a few minutes; faster and more reliable options can be suggested than conventional culture-based techniques (Sondhi et al., 2024). Generally, they utilize AgNPs as signal amplifiers in surface plasmon resonance or electrochemical detection systems, which, in turn, present robust tools for food safety monitoring.

Although promising, there are several challenges to incorporating these into the food processing chain. One major challenge includes the possibility of developing microbial resistance to silver ions over time, which would therefore limit their effectiveness long term (Li & Xu, 2024). Coatings with AgNPs provide extended antimicrobial effects, and studies to date are ambiguous about how they behave if exposed repeatedly to consecutive cycles of cleaning treatments and high temperatures combined with harsh abrasiveness typically found in the food processing environment (Kumar et al., 2021). Regulating is still an issue since the acceptance of AgNP-coated equipment often calls for detailed studies on nanoparticle migration into food products and the health risks associated. Environmental issues due to the large-scale application of AgNPs also need to be focused upon. The aggregation of AgNPs from food processing waste can contaminate water and soil ecosystems, thereby disturbing microbial diversity and ecological balance. To mitigate these harmful impacts, researchers are currently exploring eco-friendly synthesis methods and recyclable AgNP coatings to minimize the impact on the environment (Manikandan et al., 2023). Future applications of AgNPs in food processing are designed for multifunctional coatings and biosensors that may integrate antimicrobial properties, self-cleaning functions, and sensitive pathogen detection. Some of the research has dealt with innovations such as smart surfaces of AgNPs-modified surfaces that can sense contamination by releasing only the antimicrobial agents when the contamination is sensed. Furthermore, strong interschool collaboration from microbiologists, materials scientists, and regulatory agencies will be paramount in surmounting such challenges regarding the safe and efficient use of AgNPs in food processing. Silver nanoparticles have great potential and are most likely to transform food processing through effective pathogen control and subsequent food safety. Their applications on antimicrobial coatings and advanced biosensors are the most strategic tools that can make possible the reduction of contaminant risks in food production environments. In the meantime, more research and studies must be conducted to overcome the resistance, safety, and environmental sustainability issues that hinder its wide-scale adoption (Jasni et al., 2021; Siritongsuk et al., 2022).

### 7.3 Emerging applications of silver nanoparticles in agriculture to control foodborne pathogens

**Fig. 4:**
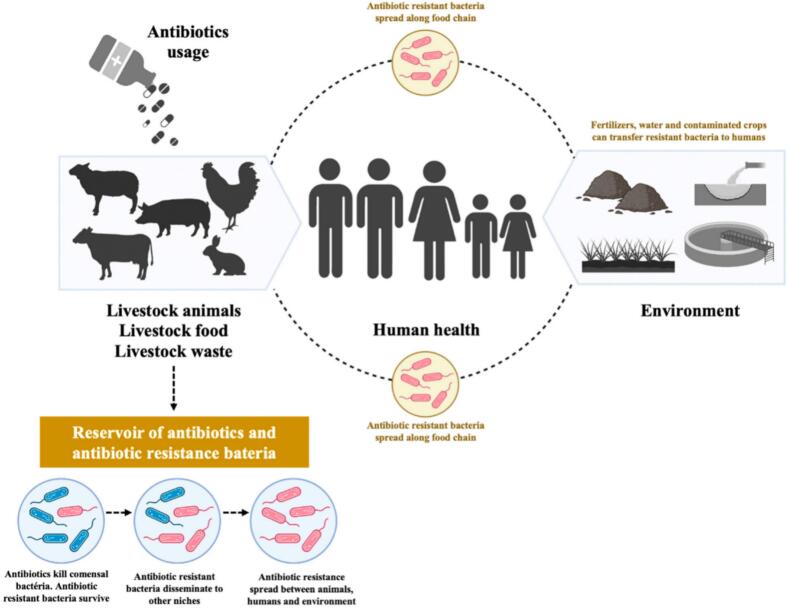
Various Route Leading to the Spread of Antibiotic-Resistant Bacteria taken from (Silva et al., 2023).

There is growing recognition that silver nanoparticles have the potential to become a new agricultural crop yield enhancer and foodborne pathogen controller. The two major nanofertilizers include antimicrobial agents that prevent microbial infections in crops and nanofertilizers that improve plant growth, facilitating nutrient uptake. These innovations are consistent with sustainable agricultural methods because they reduce chemical pesticides and fertilizers, hence improving food safety and reducing risks of contamination (Khan et al., 2023a). AgNP-based nano-fertilizers possess great potential to optimize nutrient delivery and plant growth. The nano-level size allows nanoparticles to penetrate deeper into plant tissues and root systems, thus promoting improved nutrient uptake (Mittal et al., 2020). Silver nano-fertilizers increased the uptake of nitrogen and photosynthesis efficiency of crops like wheat and maize and thus significantly impacted yield production (Khan et al., 2023b). AgNPs also have antimicrobial activity that protects plants from pathogenic pathogens that cause root diseases and promote growth in plants. Nano-fertilizers obviate the drawbacks of nutrient leaching and runoff, which are commonly associated with traditional fertilizers, to provide nutrients in a slow-release and controlled mechanism to avoid environmental pollution and maximize the utilization of available resources (Wahab et al., 2024). This two-pronged benefit from AgNPs makes them an excellent innovation for regions plagued by the issues of soil degradation and scarcity of water. In fact, very critically important to crop protection against bacterial and fungal pathogens is the antimicrobial properties of AgNPs. AgNPs degrade the microbial cell membranes and metabolic processes and effectively inhibit the growth of pathogens such as *Pseudomonas syringae, Xanthomonas campestris,* and *Fusarium oxysporum,* which cause sizeable agricultural losses (Trzcińska-Wencel et al., 2023). The sprays of AgNP on the leaves and fruits of plants are seen to create a protective barrier, reduce microbial colonization, and prolong shelf life (Noga et al., 2023). For example, Khorasani et al., 2022 demonstrated that saffron tepal-reduced AgNPs were used for the management of bacterial blight in rice. AgNPs are also applied as seed and soil treatments to avoid contamination by pathogens before harvesting, which may lead to foodborne diseases in later stages (Khorasani et al., 2022). Silver nanoparticle coatings on seeds ensure germination without pathogens. Amendments of the soil with AgNPs also inhibit soil-borne harmful micro-organisms. However, great care should be taken to avoid interference with the soil microorganisms used in nutrient cycling and overall soil health (Kim et al., 2024). Fig. 4 depicts various routes leading to the spread of antibiotic-resistant bacteria (Pérez-Rodríguez & Mercanoglu Taban, 2019; Schrijver et al., 2018; Silva et al., 2023).

Besides their application toward preventing the deterioration of crops, AgNPs are used as post-harvest treatment aimed to reduce the contamination burden and rot. Applied at the time of harvesting, the AgNPs-based coating limits the growth of pathogens on these products during the storage and transport phases, thus reducing post-harvest losses (Yan & Chen, 2019). For instance, it has been reported that the treated tomatoes and cucumbers with AgNPs maintained good quality and greatly reduced loads of microorganisms over extended periods of storage (Begum et al., 2022). Despite this, there are several issues related to the massive application of AgNPs in agriculture. One of the major concerns is the ecotoxic impact of silver nanoparticles, especially in soils and water bodies. However, AgNPs tend to accumulate in the environment and will affect the nontarget organism populations, including beneficial soil microbe populations and aquatic life. Khorasani et al., 2022 have thus emphasized the need for controlled application methods and rigorous environmental impact assessments (Khorasani et al., 2022). Another emerging concern is the problem of microbial resistance to silver that will be developed, which may further reduce the long-term efficacy of AgNPs as antimicrobial agents (McNeilly et al., 2021). Furthermore, the presence of regulatory barriers and the substantial expenses associated with the production of nanoparticles constrain their availability to smallholder farmers, who constitute a critical component of the agricultural sector in numerous developing nations. The prospective role of AgNPs in agriculture depends on the advancement of sustainable and economically viable synthesis methods that leverage renewable resources, including plant extracts or agricultural by-products. Techniques for green synthesis not only lower production costs but also mitigate the environmental hazards linked to traditional chemical synthesis approaches (Ying et al., 2022). Functionalization with other kinds of nanomaterials, for example, zinc oxide or titanium dioxide, would be able to increase functionality while widening the spectrum of antimicrobial activity. Further improvement in application toward agriculture is research into smart delivery systems, controlled-release formulations, and nanocarriers (Aguda & Lateef, 2022). Such an approach would ensure that the application was as fine-tuned as possible while at the same time minimizing harmful environmental impacts with maximum efficacy. Interdisciplinary collaboration by materials scientists, agronomists, and environmentalists can help in overcoming the challenges facing the field and ensure sustainable use of AgNP-based technologies in agriculture. Silver nanoparticles have so much potential to make the agricultural world deliver higher crop productivity and reduce microbial contamination at both pre-harvest and post-harvest levels. Their applications as nano-fertilizers and antimicrobial agents represent a step toward sustainable agriculture. However, high acceptance of AgNPs requires overcoming questions related to environmental safety and financial issues along with assurance of acceptance. Critical research and innovations for AgNPs in agricultural practice and enlightening their contribution to global food security should take place.

### 7.4 Silver nanoparticles in livestock feed for reducing gastrointestinal pathogens

Silver nanoparticles (AgNPs) are emerging as a novel additive in livestock feed, offering significant potential to reduce gastrointestinal pathogens, enhance gut health, and improve overall livestock productivity. Due to their broad antimicrobial action, AgNPs significantly inhibit pathogenic bacteria such as *Escherichia coli, Salmonella enterica,* and *Clostridium perfringens,* with minimal harm to the beneficial intestinal microbiota. This selective action is through mechanisms of disruption of bacterial cell walls, enzymatic activity inhibition, and the production of reactive oxygen species (ROS), making them an alternative effective replacement for conventional antibiotics that usually lead to resistance (De Silva et al., 2021). AgNPs have been added to animal diets, which exhibited several health benefits. Intestinal morphology was improved with increases in villus height, and increased nutrient absorption in broilers has been reported upon supplementation with AgNPs. These changes enhance feed conversion ratios and efficiency of gain, as demonstrated by an experiment in growing broiler chickens that realized significantly enhanced body weight and feed utilization in diets supplemented with AgNPs (El-Abd et al., 2022). Reduced loads of pathogens, including intestinal populations of *E. coli* and *Salmonella,* confirm the involvement of AgNPs in maintaining gut health and the beneficial microbiota (Campos et al., 2021). AgNPs economically deliver substantial benefits of reducing reliance on conventional antibiotics and loss of productivity associated with the disease.

Stability and shelf life with these materials offer stability and shelf life for which they become desirable in formulations for animal feed, especially in intensive agricultural practices. The relevant application of AgNPs also addresses the ever-rising global issue that has been drawing much attention concerning the problem of antimicrobial resistance which offers a sustainable approach toward the management of livestock health (Patra, 2019). The environmental impacts of AgNPs in animal feed must be considered in depth. Residual nanoparticles can have hazardous effects on terrestrial and aquatic ecosystems due to their incidence in manure and agricultural runoff. In this context, green synthesis methods involving materials that are biocompatible and biodegradable are also being researched to ensure ecological safety (Dang et al., 2021; Ihtisham et al., 2021). The future use of AgNPs in animal nutrition is believed to depend on the optimization of doses and delivery mechanisms that maximize benefits and minimize risks. AgNPs with prebiotics or probiotics offer promising avenues to achieve synergy in gut health. Once the AgNPs are proven safe, and production becomes eco-friendly, it may play a transformative role by bringing about an improvement in livestock productivity along routes of reducing pathogenic loads and investment in sustainable farming practices.

## 8 Regulations for the use of silver nanoparticles in the food and agriculture industry

Existing guidelines for the fields of agriculture and food require adaptation or the development of new ones, given the expansive influence of nanotechnology. Nanotechnology is a multidisciplinary domain, drawing from biology, physics, chemistry, material science, medicine, and engineering. Its interdisciplinary nature holds immense potential for advancing products, technology, and scientific knowledge (Prasad et al., 2017). In agriculture, nanotechnology is being leveraged to create advanced insecticides, boost food crop yields, and enhance biocontrol methods against various infections that threaten agricultural output. This innovative approach heralds a new era characterized by increased effectiveness, cost-efficiency, and reduced environmental and human health risks. Its applications span multiple facets of agriculture and food-related domains, including the improvement of feed and food additives, the development of sophisticated food packaging solutions, the deployment of potent antimicrobial agents for animal infection control, and, notably, the exploration of alternative strategies to address the mounting challenge of multidrug resistance in human health, resulting from excessive antibiotic use in agriculture (Yadav et al., 2019).

In the European context, regulations have indirectly addressed the utilization of nanomaterials in various applications related to food and agriculture. Regulation No 178/2002 establishes laws and regulations concerning food and feed safety in European countries, encompassing food law and addressing aspects such as food storage, and safety throughout production, processing, and transport. Under this regulation, the European Food Safety Authority (EFSA) plays a crucial role in providing scientific counsel and managing food safety issues, particularly through the RASFF Rapid Alert system for feed and food (Amenta et al., 2015a).

The term ‘Nano Foods’ is defined under Regulation (EU) 2015/2283 as novel foods, encompassing food products that incorporate nanomaterials or employ nanotechnology tools or processes across the entire food cycle, from cultivation to final consumer packaging. Any food containing engineered nanomaterials, materials with sizes of 100 nm or less displaying functional properties, or properties resembling nanomaterials, must be indicated in the ingredient list for final consumer consumption under Regulation (EU) No 1169/2011 (Radley-Gardner et al., 2016).

Furthermore, Regulation (EC) No 1333/2008 deals with food additives, including modifications involving nanotechnology, which necessitate rigorous safety assessments by EFSA before being approved for use as food additives. These regulations also apply to foods intended for children, special medical purposes, or dietary replacements for various health conditions under Regulation (EU) No 609/2013. Commission Regulation (EU) No 10/2011 addresses the utilization of nanoparticles in contact materials that come into contact with food. It emphasizes toxicological assessments and safety evaluations concerning nanoparticle composition and concentration compared to other materials used in packaging (Radley-Gardner et al., 2016).

More recently, in August 2021, the European Food Safety Authority released guidelines related to the regulation of nanomaterials intended for use in food and feed. These guidelines outline parameters for nanoparticle characterization and risk assessment factors, including exposure and hazard identification, for human and animal consumption (Committee et al., 2021).

In the United States, the utilization of nanoparticles in diverse food and agricultural contexts is subject to regulation by the US FDA. The FDA has formulated directives outlining the incorporation of nanomaterials in the food sector and their influence on the safety and regulatory standing of food components, encompassing substances in contact with food. Moreover, supplementary guidance has been furnished under the heading ‘Evaluating the consequences of substantial modifications in manufacturing processes, encompassing emerging technologies, on the safety and regulatory status of food components and substances in contact with food, including those that function as color additives (Ferraris et al., 2021).

In other countries such as Japan, specific regulations on nanotechnology and nanomaterials in the food and agriculture sectors remain unclear. China's National Centre for Nanoscience and Technology and the Commission on Nanomaterial Standardization oversee nanomaterial use, but comprehensive limits and types of nanomaterials are not specified. Australia and New Zealand have established Food Standards Australia and New Zealand (FSANZ) as an independent organization to regulate nanomaterials as food ingredients and packaging materials (Amenta et al., 2015a).

A list of regulations proposed by different countries has been listed in Table 4. Despite the regulations in different countries, specifics regarding nanoparticles, especially silver nanoparticles, remain unclear. In some European countries, silver nanoparticles are used in packaging and food contact materials, with guidelines stipulating limits of less than 0.05 mg/l in water and 0.05 mg/kg in food products (Trotta et al., 2023).

**Table 4:** . Regulations for the use of silver nanoparticles/nanomaterials in the food and agriculture industry.

**Country/Region**	**Regulatory Body**	**Food Industry Regulations**	**Agriculture Regulations**	**Key Applications**	**Notes**	**Reference**
United States	FDA, EPA	It requires safety assessments for food contact materials; nanosilver is allowed in antimicrobial packaging.	Regulated under pesticide laws; nanosilver-based fertilizers require EPA approval.	Food packaging, pest control	Emphasis on environmental safety and human health.	(Kumari et al., 2023)
European Union	EFSA, ECHA	Strict limits on nanoparticle size (<10 nm) and concentrations; mandates product labeling.	Controlled under EU pesticide laws; approval is needed for specific uses.	Pesticides, biodegradable coatings	Focus on consumer safety and reducing environmental impact.	(Nielsen et al., 2023; Singh et al., 2024)
Japan	Ministry of Health, Labour and Welfare	Requires safety evaluations for novel food additives containing nanoparticles.	Strict pre-approval for agricultural chemicals with nanosilver.	Advanced nanotechnology research	Strong emphasis on innovation and public safety.	Peters et al., 2016)
China	CFSA, Ministry of Agriculture	Mandates detailed safety evaluations; growing interest in regulating nanosilver applications.	Regulated under agricultural safety laws; environmental impact assessments are needed.	Food storage, fertilizers	Rapidly evolving regulations to meet international standards.	(“China Notifies for Safety Evaluation Materials for “Three New Foods” [WWW Document, 2024)
Australia	FSANZ, APVMA	Requires pre-market approval for food products with nanomaterials.	Nanosilver fertilizers are regulated as agricultural chemicals; safety data is required.	Veterinary products, food coatings	Strong focus on consumer and environmental protection.	(“Australian Pesticides and Veterinary Medicines Authority, 2010)

## 9 Commercial feasibility of silver nanoparticles for foodborne infection applications

The commercial feasibility of AgNPs in controlling foodborne infections is based on patent development, production scaling up, cost reduction, and the dismantling of obstacles to marketplace adoption. Innovation in the patent landscape is significant concerning green synthesis techniques involving biological reducing agents through plant-based synthesis agents, antimicrobial food packaging, and direct applications on the surface of food. For example, US10292538B2 details are friendly to the environment synthesis, while EP2917850A1 only suggests that the packaging films should be AgNP-based and, as such need to show inhibition of microbial growth to attain and extend shelf life (Lawal et al., 2024). Still, difficulties lie in scaling up to industrial-scale production; achieving homogeneity of size and shape is necessary and important for efficacy. Scaling production to industrial levels presents challenges, including maintaining uniformity in particle size and shape, which are critical for antimicrobial efficacy. Studies such as (Lisboa et al., 2024) highlight the importance of precise production controls to minimize variability, while innovations like biogenic synthesis using agricultural waste offer cost-effective and sustainable alternatives (Larrañaga-Tapia et al., 2024). However, mass production requires substantial capital expenditure using specific machinery and procedures (Gupta et al., 2024). Similarly, aerosol-based synthesis methods are promising for scale-up production with economies of cost due to their scalability feature (Fisher et al., 2023).

One of the factors considered in the production cost of silver nanoparticles (AgNPs) is the high price of silver as the primary raw material. Efforts to alloy AgNPs with other alternative metals have been shown to have great potential for cost-saving (Singh et al., 2023). Energy-efficient and environment-friendly synthesis techniques have been investigated in an attempt to cut down the operational costs (Rosman et al., 2021). Realizing economies of scale via advanced production technologies is crucial for lowering per-unit expenses and enhancing the competitiveness of these materials within the marketplace. Notwithstanding these developments, numerous obstacles hinder extensive market integration. Regulatory hurdles continue to be considerable, with differing standards among nations prolonging the endorsement process for AgNP-based food safety solutions (Vidic et al., 2019). Furthermore, apprehensions related to toxicity and environmental repercussions require comprehensive safety evaluations (Cerqueira et al., 2018). Consumer acceptance is another problem where public awareness of the use of nanotechnology in food safety is still at the infant stage. With proper education and labeling, it can establish comprehension and faith among consumers (Mohanty et al., 2023). The future of AgNPs in the control of foodborne infection will only lie in continued innovation and concerted work between researchers, regulatory agencies, and industry stakeholders. Addressing the issues of scalability, sustainability, and safety, AgNPs are likely to emerge as a novel solution for improved food safety with very minimal microbial contamination. Researchers like Lisboa et al., 2024 and Rosman et al., 2021 allow such barriers to be reduced and for the full potential of this promising technology toward its full commercial development to be realized. Lastly Table 5 lists silver nanoparticle (AgNP)-based products and their applications in the antimicrobial control of food-borne pathogens.

**Table 5:** . Overview of various silver nanoparticle (AgNP)-based products and their applications in antimicrobial control of food-borne pathogens.

**Product/Brand**	**Application**	**Mechanism**	**Manufacturer/Source**	**Regulatory Status**	**Target Microorganisms**	**Reference/Source**
**NanoCid®**	Antimicrobial coating and sprays	Bactericidal action on surfaces	NanoCide Technologies	Approved in some regions; under review elsewhere	*E. coli*, *Listeria monocytogenes*, *Salmonella*	(Zarei et al., 2014)
**AgNP-based Biodegradable Films**	Food packaging for poultry and meats	Inhibits microbial growth; biodegradable matrix	Academic R&D collaboration	Pre-commercial prototype	*Salmonella*, *Campylobacter jejuni*	(das Neves et al., 2023)
**Green-Synthesized AgNPs**	Surface sprays against *Salmonella*	Disruption of bacterial cell walls	Biogenic Synthesis, Independent Labs	In development	*Salmonella*, *E. coli*, *S. aureus*	(Losasso et al., 2014)
**Essential Oil-AgNP Combinations**	Pathogen control in packaged food products	Synergistic antibacterial and antifungal effects	R&D Academic-Industry Partnerships	Not yet commercialized	*Candida albicans*, *E. coli*, *Aspergillus*	(Begum et al., 2022)
***Terminalia Catappa*-derived AgNPs**	Combat biofilms in food industries	Biofilm penetration and bacterial inhibition	Terminalia Research Unit	Under regulatory evaluation	*Listeria monocytogenes*, biofilms	(Devadiga et al., 2017)
**Campylobacter-targeted AgNPs**	Antimicrobials in poultry processing	Interference with bacterial metabolism	Research Collaboration, Mexico	Early-stage regulatory review	*Campylobacter jejuni*	(Rivera-Mendoza et al., 2024b)
**Valorized AgNPs from Saffron Tepals**	Poultry product pathogen control	Eco-friendly synthesis and strong bactericidal effect	Academic Labs	Pilot-scale testing	*E. coli*, *Salmonella*, *Pseudomonas aeruginosa*	(Khorasani et al., 2022)

## 10 Conclusion

Silver nanoparticles have immense transformative potential in addressing foodborne infections and antimicrobial resistance, and it has versatile applications in food packaging, processing, agriculture, and livestock management. Its unique multi-targeting mechanism of antimicrobial action, broad-spectrum efficacy, and synergetic action with classical antibiotics make this a very powerful tool for the improvement of food safety. Furthermore, the sustainability, scalability, and cost-effectiveness of green synthesis routes of production have significantly improved AgNP production against global goals of sustainability. However, there are enough implications about the environmental and ecotoxicological impact of AgNPs, including their interference with soil and aquatic ecosystems and bioaccumulation in food chains, so systemic safety appraisal and proper legislative framework should be built. Interdisciplinary academic contributions between scientists, policymakers, and industry stakeholders could be used to better optimize AgNP applications, mitigate risks, and build consumer trust as regards the potential of AgNPs. This is possible through AgNPs as they bridge knowledge gaps and foster innovation, hence revolutionizing approaches to food safety as a sustainable and effective strategy for combating foodborne pathogens that would safeguard global health.

## CRediT authorship contribution statement

**Himanshu Jangid:** Writing – original draft. **Harish Chandra Joshi:** Writing – review & editing, Writing – original draft. **Joydeep Dutta:** Writing – original draft, Conceptualization. **Akil Ahmad:** Writing – original draft. **Mohammed B. Alshammari:** Writing – review & editing, Supervision. **Kaizar Hossain:** Writing – review & editing, Supervision, Conceptualization. **Gaurav Pant:** Writing – review & editing, Supervision, Conceptualization. **Gaurav Kumar:** Writing – review & editing, Supervision, Conceptualization.

## Declaration of competing interest

The authors declare that they have no known competing financial interests or personal relationships that could have appeared to influence the work reported in this paper.

## Data Availability

No data was used for the research described in the article.
